# Noninvasive Preoperative Stratification of Renal Tumors for Aggressiveness and Prognosis

**DOI:** 10.3390/bioengineering13091084

**Published:** 2026-09-19

**Authors:** Jinglai Lin, Linpeng Yao, Jianbo Gao, Ning Xu, Ying Xiong, Qi Bai, Kang Wang, Qi Sun

**Affiliations:** 1 Department of Urology, Zhongshan Hospital (Xiamen), Fudan University, Xiamen 361015, China; linjinglainbk@gmail.com (J.L.); xiong.ying@zs-hospital.sh.cn (Y.X.); 2 Xiamen Clinical Research Center for Cancer Therapy, Zhongshan Hospital (Xiamen), Fudan University, Xiamen 361015, China; 3 Clinical Research Center for Precision Medicine of Abdominal Tumor of Fujian Province, Zhongshan Hospital (Xiamen), Fudan University, Xiamen 361015, China; 4 Department of Urology, Urology Research Institute, The First Affiliated Hospital, Fujian Medical University, Fuzhou 350005, China; 5 Department of Radiology, The First Affiliated Hospital, Zhejiang University School of Medicine, Hangzhou 310000, China; 6 Department of Urology, The People’s Hospital of Lincang, Lincang 677000, China; 18787251389@163.com; 7 Department of Urology, Zhongshan Hospital, Fudan University, Shanghai 200000, China; bai.qi@zs-hospital.sh.cn; 8 Digital Medical Research Center, School of Basic Medical Sciences, Fudan University, Shanghai 200000, China; 9 Department of Pathology, Zhongshan Hospital (Xiamen), Fudan University, Xiamen 361015, China

**Keywords:** renal tumors, aggressiveness, computed tomography, radiomics, prognosis

## Abstract

Accurate preoperative discrimination between indolent and aggressive renal lesions is essential for personalized risk-stratified management and improves the clinical outcomes of renal tumor patients. This study aimed to establish and externally validate a non-invasive computed tomography (CT)-derived radiomic model for identifying aggressive renal tumors and predicting long-term postoperative survival. A total of 3407 patients with pathologically confirmed renal tumors from multiple centers were retrospectively enrolled and divided into three independent cohorts: a training cohort (*n* = 1840), an internal validation cohort (*n* = 789), and an external test cohort (*n* = 778). Based on preoperative multiphasic CT images, a support vector machine (SVM)-based radiomic model was constructed to evaluate tumor aggressiveness. Cox proportional hazards regression analysis was performed to identify independent prognostic factors, and a comprehensive nomogram combining radiomic aggressiveness score and clinicopathological parameters was further developed for survival prediction. The established SVM radiomic model yielded stable discriminatory performance, with an area under the receiver operating characteristic curve (AUC) of 0.740 in the internal validation cohort and 0.763 in the external test cohort. Multivariate Cox regression analysis demonstrated that radiomics-predicted tumor aggressiveness remained an independent adverse prognostic factor for recurrence-free survival (HR = 3.656), overall survival (HR = 1.981), and disease-specific survival (HR = 3.673). Moreover, the integrated nomogram demonstrated favorable calibration for predicting 5-year and 8-year overall survival in the internal validation cohort. This novel CT-based radiomic model provides a robust non-invasive tool for preoperative differentiation of renal tumor aggressiveness. As an independent prognostic biomarker, it can assist clinicians in precise preoperative risk evaluation and facilitate individualized therapeutic decision-making for renal tumor patients.

## 1. Introduction

Renal cell carcinoma (RCC) is a common urological malignancy originating from the renal tubular epithelium. In 2022, there were approximately 435,000 new cases and 156,000 deaths globally, accounting for 2–3% of all adult malignancies, with a higher incidence in developed countries compared to developing nations [1,2,3]. According to the 2024 data from China’s National Cancer Center, the incidence of RCC in China has been increasing annually, with approximately 74,000 new cases and 24,000 deaths. China currently has one of the highest numbers of RCC incidence and mortality worldwide, and its mortality-to-incidence ratio (0.39–0.58) is significantly higher than the global average (0.39) and that of the United States (0.17) [3,4], highlighting a severe disease burden and an urgent need to improve early screening and diagnostic systems. In recent years, with the widespread application of cross-sectional imaging modalities such as abdominal CT and MRI, an increasing number of asymptomatic, localized renal masses, particularly small renal masses (SRMs), are being incidentally detected during routine physical examinations or screening for other conditions [5]. However, localized renal tumors are not a homogeneous entity; they exhibit significant variations in pathological composition and biological behavior, presenting either as relatively indolent lesions or as highly aggressive tumors with substantial progression potential. Given this background, the clinical management of localized renal tumors has gradually shifted from a uniform surgical resection approach toward more individualized, risk-adapted strategies, including nephron-sparing surgery, thermal ablation, and active surveillance [5,6].

Consequently, the focus of preoperative assessment is no longer limited to a binary “benign versus malignant” classification but emphasizes the identification of the tumor’s biological aggressiveness. For selected patients who are of advanced age, have multiple comorbidities, or present with small and radiographically stable tumors, active surveillance has become a clinically acceptable management pathway. Conversely, lesions with high potential aggressiveness require more proactive intervention strategies [5,7]. Therefore, a more accurate pretreatment differentiation between indolent and aggressive renal tumors would facilitate optimal selection among active surveillance, local ablation, nephron-sparing surgery, and radical resection, thereby minimizing overtreatment while avoiding the underestimation of high-risk lesions [5,6,7].

Currently, contrast-enhanced CT and MRI remain the core imaging tools for the diagnosis, staging, and treatment decision-making of renal tumors. However, traditional visual interpretation of imaging has distinct limitations in recognizing tumor aggressiveness, particularly when there is a high degree of overlap among different histological subtypes, nuclear grades, and intratumoral heterogeneity [5,7]. Although renal mass biopsy (RMB) can provide preoperative pathological information and has demonstrated favorable safety and diagnostic efficacy in recent years, its application remains selective. Furthermore, inherent issues such as sampling error, tumor heterogeneity, and restricted capability in assessing tumor grade and adverse pathological features continue to limit its universal implementation across all patients [8,9]. Therefore, developing a non-invasive, quantitative assessment method based on routine preoperative imaging to reflect tumor biological behavior is of critical clinical significance.

The evolution of radiomics and artificial intelligence (AI) has provided a novel technical paradigm for the non-invasive and precise evaluation of renal tumors. By extracting high-throughput quantitative features from routine medical images, radiomics can characterize tumor heterogeneity to a considerable extent and has been utilized for RCC nuclear grading prediction, recurrence risk assessment, and survival modeling [10,11]. Recent studies have indicated that CT radiomics holds promising potential in predicting the WHO/ISUP grade of clear cell RCC, postoperative recurrence risk, and overall survival. Nevertheless, existing evidence predominantly focuses on the grading of clear cell RCC or risk stratification within a single histological subtype, with insufficient attention paid to a comprehensive “indolent/aggressive” phenotype that better reflects real-world clinical decision-making scenarios [12,13].

Emerging research further demonstrates that AI models based on preoperative multiphase CT can not only non-invasively identify the pathological features of renal tumors, but also distinguish between indolent and aggressive phenotypes, which correlates with disease-specific survival, recurrence-free survival, and overall survival outcomes [14]. This suggests that radiographically predicted “aggressiveness” may serve not merely as a diagnostic label, but as a preoperative biological phenotype carrying prognostic significance. Therefore, this study aimed to develop and validate a preoperative CT-based radiomics model to differentiate indolent from aggressive renal tumors, and to further investigate the association between model-predicted aggressiveness and postoperative survival outcomes. Ultimately, this approach seeks to provide non-invasive imaging evidence for precise preoperative risk stratification and individualized treatment decision-making in patients with renal tumors.

## 2. Materials and Methods

**Patient selection**

This study retrospectively included patients who underwent partial or radical nephrectomy at Zhongshan Hospital of Fudan University, First Affiliated Hospital of Zhejiang University School of Medicine, First People’s Hospital of Lianyungang, Xiamen Branch of Zhongshan Hospital Fudan University, Affiliated Zhongshan Hospital of Dalian University, and Zhangye People’s Hospital between 2009 and 2022. Inclusion criteria required pathologically confirmed renal tumors with complete preoperative unenhanced, arterial, and venous phase CT imaging. Patients with incomplete imaging or pathological data, imaging artifacts precluding analysis, pathologic N1 disease, AJCC stage IV disease, or receipt of neoadjuvant systemic therapy before surgery or adjuvant systemic therapy after surgery were excluded. Only malignant cases with follow-up information were included in the survival analysis. A total of 2629 patients from Zhongshan Hospital of Fudan University, First People’s Hospital of Lianyungang, Xiamen Branch of Zhongshan Hospital Fudan University, Affiliated Zhongshan Hospital of Dalian University, and Zhangye People’s Hospital were randomly divided into a training cohort (*n* = 1840) and an internal validation cohort (*n* = 789) at a 7:3 ratio. Additionally, 778 patients from the First Affiliated Hospital of Zhejiang University School of Medicine served as an independent external test cohort. This study complied with the Declaration of Helsinki and was approved by the institutional review boards of all participating hospitals.

**Histologic classification**

Renal tumors were stratified into indolent and aggressive tumors based on their histopathological characteristics. The indolent group comprised clear cell renal cell carcinoma (ccRCC) and papillary RCC (pRCC) devoid of major vein or perinephric tissue invasion, grade 3–4 components, or sarcomatoid differentiation, with the additional requirement of no necrosis for ccRCC. This category also included chromophobe RCC, clear cell papillary renal cell tumors, multilocular cystic renal neoplasms of low malignant potential, succinate dehydrogenase-deficient RCC, mucinous tubular and spindle cell carcinoma, tubulocystic RCC, nephroblastoma, epithelioid angiomyolipoma, solitary fibrous tumors, well-differentiated neuroendocrine tumors, and other oncocytic neoplasms. Conversely, the aggressive group encompassed non-indolent ccRCC and pRCC, TFE3-rearranged RCC, collecting duct carcinoma, medullary carcinoma, and RCC not otherwise specified (NOS), alongside primary renal sarcomas such as leiomyosarcoma, rhabdomyosarcoma, synovial sarcoma, and Ewing sarcoma. Notably, any histological subtype exhibiting sarcomatoid differentiation was strictly classified as aggressive regardless of its primary diagnosis. The above histopathological classification served as the reference standard for model training and evaluation. WHO/ISUP grading was considered applicable to ccRCC and pRCC. ccRCC or pRCC cases without an available grade were categorized as “Unknown”, whereas histologic subtypes for which WHO/ISUP grading was not applicable were categorized as “Not applicable”. For subsequent statistical analyses, these patients were combined into a single “Unknown/Not applicable” category.

**ROI Acquisition and Refinement**

To achieve high-fidelity segmentation of the renal lesions, we implemented a hybrid strategy combining automated deep learning with rigorous manual expert verification. Initially, a model based on the nnU-Net architecture—pre-trained on the KiTS dataset—was fine-tuned using a dedicated subset of 100 arterial-phase scans from our institutional repository. For this training subset, the kidney and tumor boundaries were manually delineated slice-by-slice in the axial plane by an abdominal radiologist with four years of experience using 3D Slicer (version 5.2.2). This manually annotated dataset served as the reference standard for model optimization. The resulting model achieved a robust Dice similarity coefficient (DSC) of 0.852 in internal validation, demonstrating high consistency with expert delineations. Following the automated processing of the remaining cohort, a quality control phase was conducted: the aforementioned radiologist reviewed all generated ROIs and performed manual corrections where necessary to guarantee high-quality annotations. Finally, non-contrast and venous-phase images were spatially aligned to the arterial-phase reference using 3D Slicer’s general registration tool, ensuring that the ROIs accurately and consistently encompassed the tumor volume across all three imaging stages.

**Radiomics Feature Extraction and Selection**

Radiomics features were systematically extracted from the three CT phases using the PyRadiomics library (version 3.0.1) in Python. Pre-processing involved resampling all images to a consistent isotropic resolution of 1.0 × 1.0 × 1.0 mm^3^, with all other parameters maintained at default settings optimized for CT data. Feature extraction was performed on the axial slice exhibiting the maximum tumor diameter. For each patient, we computed 100 features per phase, categorized into 2D shape descriptors, first-order statistics, and five texture matrix families: Gray-Level Co-occurrence Matrix (GLCM), Gray-Level Dependence Matrix (GLDM), Gray-Level Run Length Matrix (GLRLM), Gray-Level Size Zone Matrix (GLSZM), and Neighborhood Gray-Tone Difference Matrix (NGTDM). After identifying and removing cross-phase redundant shape features, a final set of 282 radiomics features was consolidated for further dimensionality reduction and modeling.

To further identify the most informative and discriminative features, a three-step feature selection procedure was performed. First, pairwise correlations among the radiomic features were assessed using Pearson’s correlation coefficient to reduce feature redundancy. For each pair of highly correlated features (|*r*| > 0.90), one feature was removed to minimize multicollinearity and redundant information. At this stage, 178 out of the initial 282 multi-phase radiomic features were excluded, leaving 104 features for subsequent analysis. Second, univariate statistical analysis was performed on the remaining features to identify those showing significant differences between the two groups. For normally distributed continuous features, an independent-samples Student’s *t*-test was used, and features with *p* < 0.05 were retained. After excluding features without statistically significant between-group differences, 45 features remained. Finally, the least absolute shrinkage and selection operator (LASSO) was applied to further reduce feature dimensionality. The regularization parameter (alpha) was optimized to 0.0111 through an automated parameter search. Thirteen features with nonzero LASSO coefficients were retained and ranked according to the absolute magnitude of their coefficients, and the top 10 features were selected as the final feature set. All feature selection procedures were performed exclusively on the training cohort, and the resulting feature set was subsequently applied unchanged to the validation and external test cohorts.

**Radiomics Model Development**

The differentiation between indolent and aggressive renal tumors was formulated as a binary classification task. The final classification model consisted of an ensemble of five support vector machine (SVM) classifiers obtained through fivefold cross-validation. Each SVM used a linear kernel and was implemented using the scikit-learn library. The regularization parameter C was set to 1.0, and the random seed was fixed at 1 to ensure reproducibility. Specifically, the training cohort was divided into five folds, and in each cross-validation iteration, four folds were used to train an independent SVM classifier, resulting in five independently trained classifiers. During inference, each classifier generated a predicted probability for each case, and the mean probability across the five classifiers was used as the final radiomics-predicted aggressiveness score. The optimal classification threshold was determined in the internal validation cohort by maximizing the Youden index and was set at 0.4108. Cases with predicted probabilities above the threshold were classified as aggressive tumors, whereas those below the threshold were classified as indolent tumors. This strategy avoided using the external test cohort for threshold selection and minimized the risk of information leakage.

**Statistical analyses**

The compilation and descriptive statistics of baseline clinicopathological data were performed using SPSS version 26.0. Continuous variables were expressed as medians and interquartile ranges (IQRs), while categorical variables were presented as frequencies and percentages. Model construction, feature selection, and performance evaluation were conducted in the Python environment. Model discriminative performance was assessed using receiver operating characteristic (ROC) curves and the area under the curve (AUC), with binary classification metrics described by accuracy, sensitivity, specificity, and F1 score. For survival analysis, Kaplan–Meier survival curves were plotted, and the log-rank test was used to evaluate differences between groups. Univariate and multivariate analyses were performed using Cox proportional hazards regression models. To compare the discriminative ability of different predictors, the Harrell concordance index (C-index) was calculated, comparing the radiomics aggressiveness score with indicators such as TNM stage. The study endpoints for survival analysis included overall survival (OS), recurrence-free survival (RFS), and disease-specific survival (DSS). OS was defined as the time from surgery to death from any cause; RFS was defined as the time from surgery to local recurrence, distant metastasis, or tumor-related death; and DSS was defined as the time from surgery to renal tumor-related death. For the prognostic nomogram, 5-year and 8-year OS were selected as the prediction horizons. The 8-year time point was selected as the long-term prediction endpoint because the number of patients remaining at risk decreased substantially beyond 8 years, thereby limiting the stability of a 10-year calibration analysis.

All statistical tests were two-sided, with *p* < 0.05 considered statistically significant.

## 3. Results

**Baseline patient characteristics**

This study included a total of 3407 patients with surgically and pathologically confirmed renal tumors, comprising 1840 patients in the training cohort, 789 in the internal validation cohort, and 778 in the independent external test cohort. Patient inclusion and cohort allocation are shown in Figure S1, and the clinicopathological characteristics across the three cohorts are summarized in Table 1. The median ages of patients in the training, internal validation, and external test cohorts were 58.0 (IQR, 50.0–66.0), 57.0 (IQR, 50.0–65.0), and 56.0 (IQR, 47.0–64.0) years, respectively, and the corresponding proportions of male patients were 66.8%, 65.9%, and 68.6%. Partial nephrectomy was performed in 42.8%, 44.9%, and 69.2% of patients, respectively, whereas radical nephrectomy was performed in 57.2%, 55.1%, and 30.8%. The median tumor sizes were 3.5 (IQR, 2.5–5.0), 3.5 (IQR, 2.5–5.0), and 3.2 (IQR, 2.4–4.5) cm, and small renal masses (≤4 cm) accounted for 63.4%, 64.6%, and 70.8% of the three cohorts, respectively. Solid tumors accounted for 83.6%, 84.4%, and 87.7%, whereas cystic tumors accounted for 16.4%, 15.6%, and 12.3%, respectively. The proportions of pathologically aggressive tumors were 24.4%, 24.3%, and 23.1%, respectively. Clear cell RCC was the most common histologic subtype, accounting for 80.7%, 79.7%, and 82.9% of tumors in the three cohorts.

**Radiomics feature selection**

The final radiomic features used as inputs to the classifier were extracted from 2D ROIs on axial CT slices from the non-contrast (N), arterial (A), and venous (V) phases and selected using LASSO regression (λ = 0.011). Thirteen features with non-zero LASSO coefficients were retained after LASSO. These features were ranked according to the absolute magnitudes of their coefficients, and the top 10 were selected as the final feature set for classification modeling (Table S1). The selected features encompassed shape descriptors, first-order intensity statistics, and texture features derived from GLCM and GLSZM, indicating that complementary information on tumor morphology, intensity distribution, and intratumoral texture contributed to the classification. These features were distributed across the N, A, and V phases, including non-contrast shape and intensity features, arterial-phase intensity and texture features, and venous-phase intensity and texture features, suggesting that multiphasic CT provides complementary information for differentiating aggressive tumor from indolent tumor.

**Performance of the radiomics model in differentiating indolent and aggressive renal tumors**

**Figure 1:**
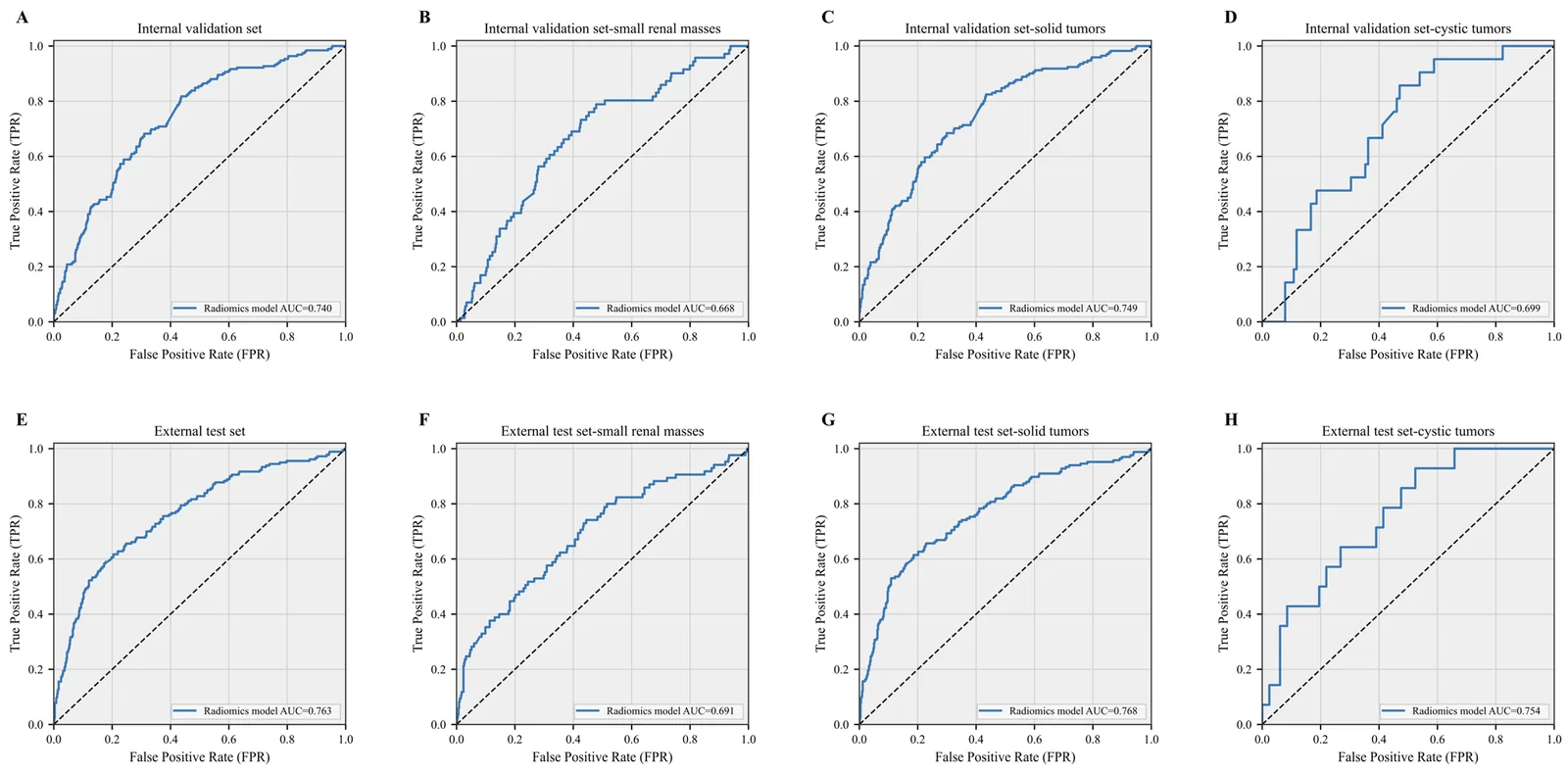
**Diagnostic performance of the radiomics model for discriminating indolent and aggressive renal tumors.** ROC curves for the overall internal validation cohort (**A**), small renal mass subgroup in the internal validation cohort (**B**), solid tumor subgroup in the internal validation cohort (**C**), and cystic tumor subgroup in the internal validation cohort (**D**). Corresponding ROC curves are shown for the overall external test cohort (**E**), small renal mass subgroup (**F**), solid tumor subgroup (**G**), and cystic tumor subgroup (**H**).

The radiomics model demonstrated consistent discriminative performance across the training, internal validation, and external test cohorts (Table 2, Figure 1). In the training cohort, the model achieved an AUC of 0.723 (95% CI, 0.699–0.752), with an accuracy of 0.596, sensitivity of 0.780, specificity of 0.537, and F1 score of 0.485. In the internal validation cohort, the AUC was 0.740 (95% CI, 0.697–0.776), with an accuracy of 0.624, sensitivity of 0.812, specificity of 0.563, and F1 score of 0.512. In the independent external test cohort, the model achieved an AUC of 0.763 (95% CI, 0.730–0.800), with an accuracy of 0.746, sensitivity of 0.617, specificity of 0.784, and F1 score of 0.529. Subgroup ROC analyses further showed AUCs of 0.668, 0.749, and 0.699 for small renal masses, solid tumors, and cystic tumors in the internal validation cohort, respectively, and corresponding AUCs of 0.691, 0.768, and 0.754 in the external test cohort (Figure 1).

**Association between radiomics-predicted aggressiveness and survival outcomes**

**Figure 2:**
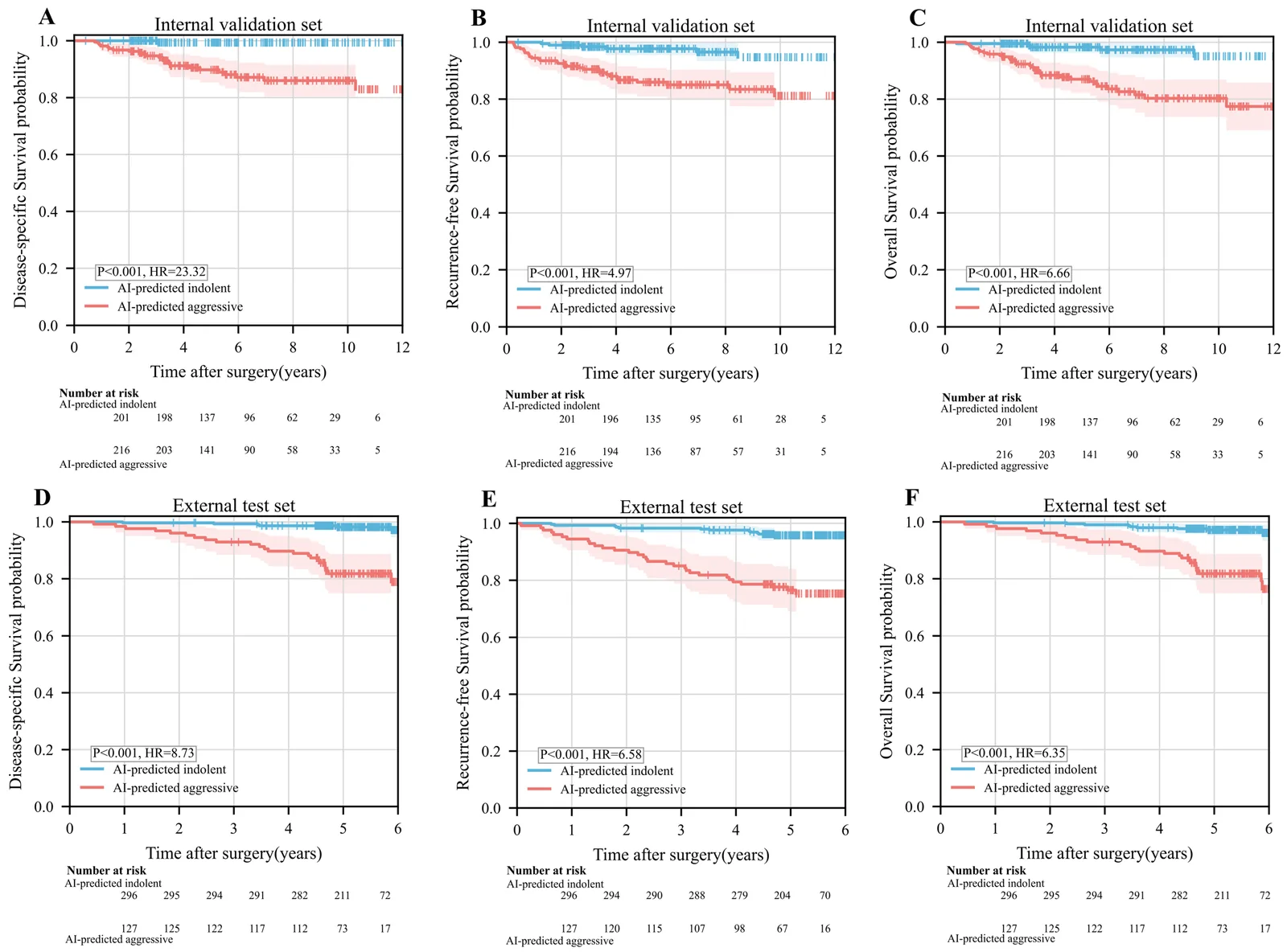
**Kaplan–Meier survival analyses according to radiomics-predicted aggressiveness.** Kaplan–Meier curves comparing disease-specific survival (DSS) (**A**), recurrence-free survival (RFS) (**B**), and overall survival (OS) (**C**) between the radiomics-predicted indolent and aggressive groups in the internal validation cohort. Corresponding DSS (**D**), RFS (**E**), and OS (**F**) analyses are shown for the external test cohort.

Kaplan–Meier survival analyses demonstrated that patients classified as radiomics-predicted aggressive had significantly poorer DSS, RFS, and OS than those classified as radiomics-predicted indolent in both the internal validation and external test cohorts (Figure 2). Consistent with these findings, Cox regression analyses showed that radiomics-predicted aggressiveness was significantly associated with adverse survival outcomes. For RFS, radiomics-predicted aggressive tumors were associated with an HR of 6.565 (95% CI, 4.353–9.902; *p* < 0.001) in univariate analysis and remained independently associated with RFS after multivariate adjustment (HR = 3.656, 95% CI, 2.362–5.659; *p* < 0.001). For OS, the corresponding univariate and multivariate HRs were 3.419 (95% CI, 2.428–4.816; *p* < 0.001) and 1.981 (95% CI, 1.361–2.882; *p* < 0.001), respectively. For DSS, the univariate HR was 7.444 (95% CI, 4.392–12.616; *p* < 0.001), and radiomics-predicted aggressiveness remained independently prognostic after multivariate adjustment (HR = 3.673, 95% CI, 2.095–6.439; *p* < 0.001). These findings indicate that radiomics-predicted aggressiveness provides prognostic information beyond its role in preoperative tumor classification (Table 3, Table S2 and Table S3, Figure 2).

**Traditional clinicopathological variables and independent prognostic factors**

Beyond radiomics-predicted aggressiveness, several traditional clinicopathological variables were also independently associated with prognosis in the updated multivariate analyses (Table 3, Table S2 and Table S3). In the RFS analysis, both TNM stage II (HR = 1.784, 95% CI: 1.138–2.797, *p* = 0.012) and stage III (HR = 3.885, 95% CI: 2.702–5.587, *p* < 0.001) remained independent adverse factors compared with stage I. WHO/ISUP grade III (HR = 4.771, 95% CI: 1.712–13.295, *p* = 0.003) and grade IV (HR = 5.225, 95% CI: 1.458–18.732, *p* = 0.011) were independently associated with poorer RFS; necrosis also retained statistical significance (HR = 1.497, 95% CI: 1.037–2.161, *p* = 0.031). In addition, sarcomatoid differentiation remained independently associated with poorer RFS after multivariate adjustment (HR = 4.414, 95% CI: 2.132–9.136, *p* < 0.001) (Table 3). In the OS analysis, TNM stages II and III, WHO/ISUP grades III and IV, necrosis, and sarcomatoid differentiation similarly remained independent adverse prognostic factors (Table S2). Similar findings were observed for DSS, with TNM stages II and III, WHO/ISUP grades III and IV, necrosis, and sarcomatoid differentiation retaining statistical significance after multivariate adjustment (Table S3). Overall, radiomics-predicted aggressiveness maintained independent prognostic significance after adjustment for TNM stage, WHO/ISUP grade, necrosis, and sarcomatoid differentiation, indicating its potential as a valuable supplement to traditional clinicopathological indicators.

**Stratification analysis and comparison of predictive discriminative ability**

**Figure 3:**
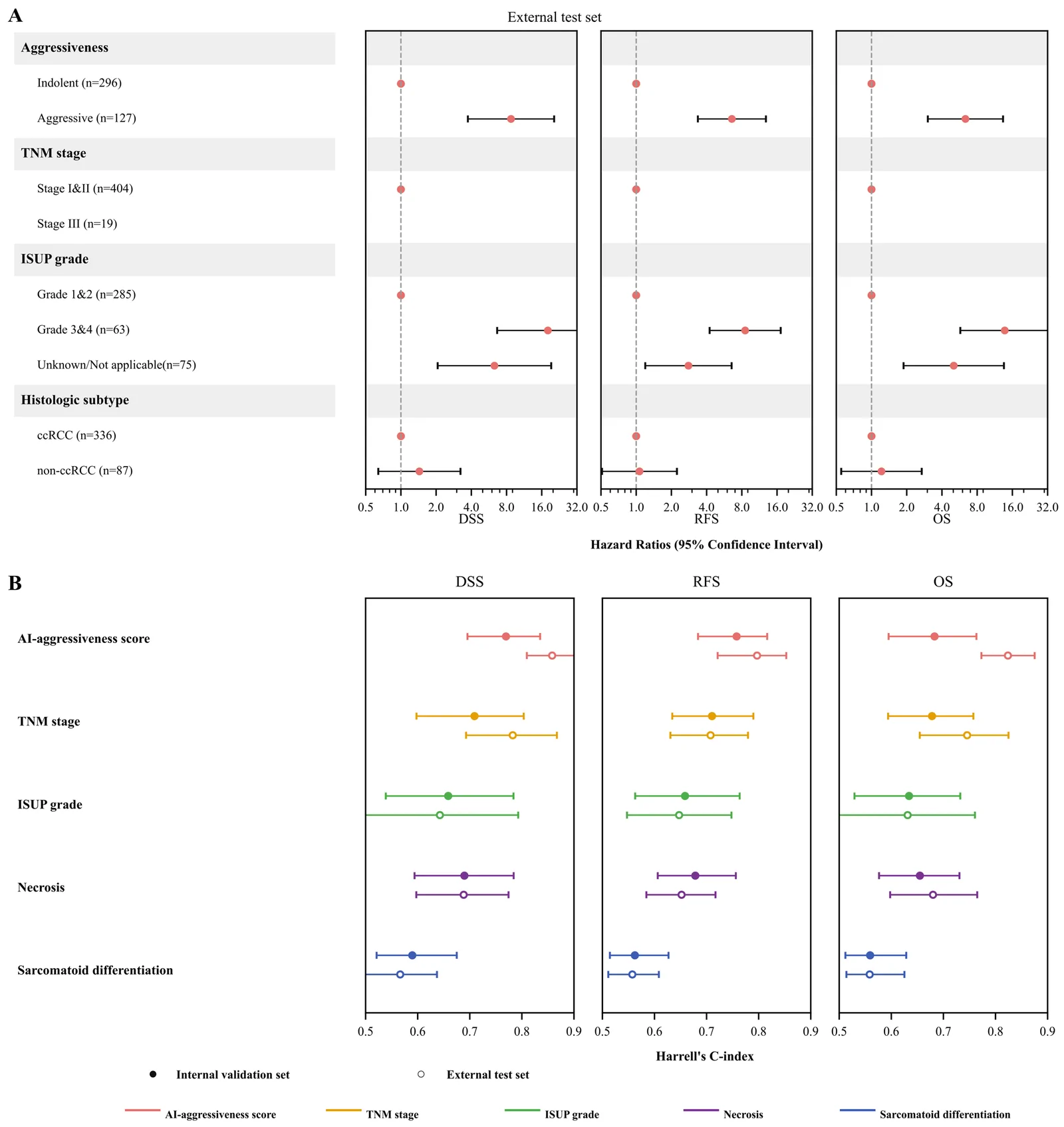
**Subgroup analyses and prognostic discrimination of radiomics-predicted aggressiveness.** Lollipop plots with 95% confidence intervals showing subgroup-specific hazard ratios for the association between radiomics-predicted aggressiveness and survival outcomes in the external test cohort (**A**). Harrell’s concordance index (C-index) comparing the prognostic performance of the radiomics aggressiveness score with conventional clinicopathologic variables, including TNM stage, WHO/ISUP grade, necrosis, and sarcomatoid differentiation, for DSS, RFS, and OS. Error bars represent 95% confidence intervals (**B**).

The association between the radiomics aggressiveness score and survival outcomes remained generally consistent across different clinicopathological subgroups, with the relevant stratification results presented in Figure 3A. Concurrently, comparisons of the Harrell concordance index revealed that the radiomics aggressiveness score outperformed individual traditional clinicopathological variables in terms of overall prognostic discrimination capability (Figure 3B). These findings further support the potential clinical utility of the radiomics aggressiveness score in preoperative risk stratification (Figure 3A,B).

**Construction and calibration of the nomogram**

**Figure 4:**
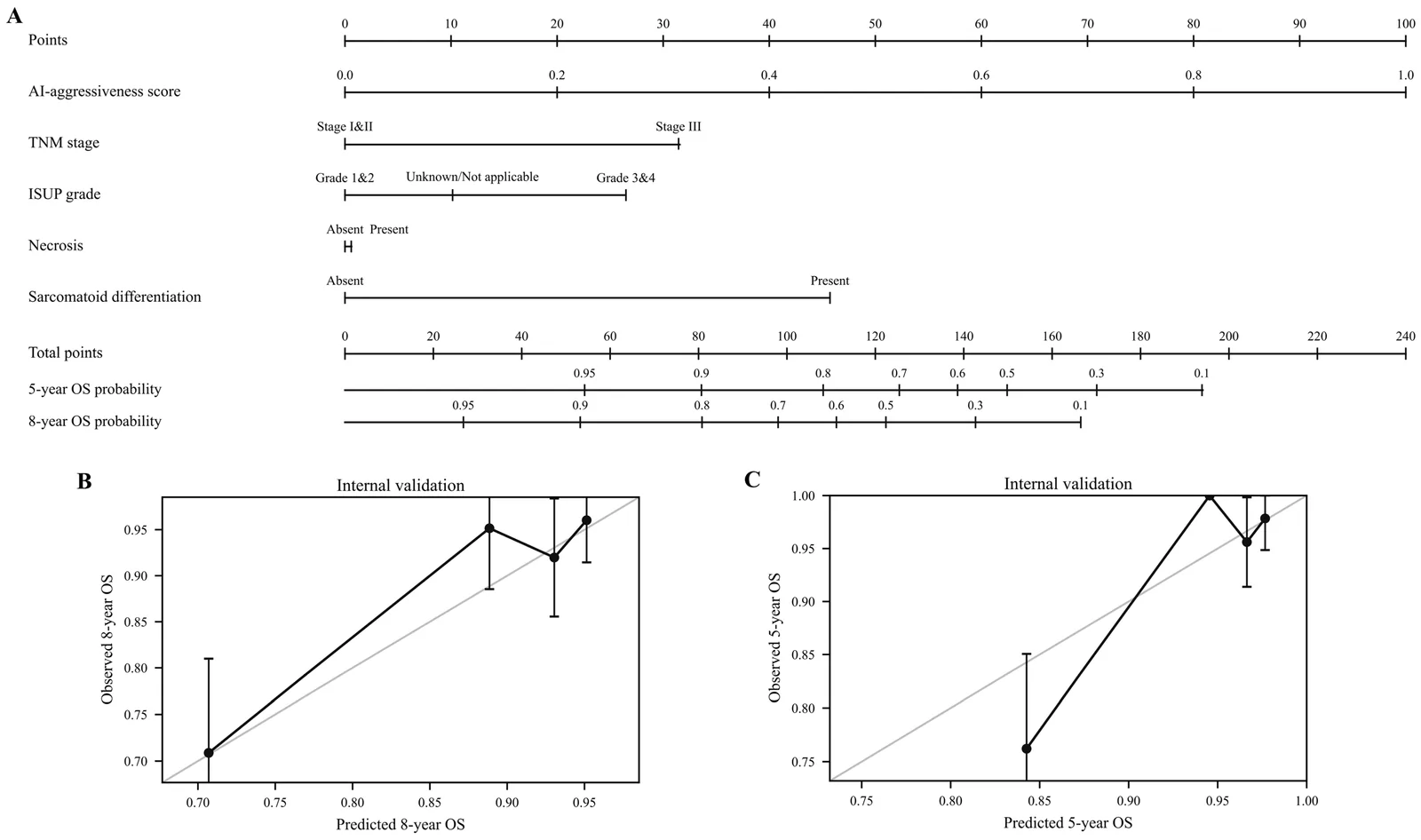
**Nomogram for predicting overall survival and calibration performance in the internal validation cohort.** Nomogram developed using the AI-aggressiveness score, TNM stage (stages I–II vs. stage III), WHO/ISUP grade, necrosis, and sarcomatoid differentiation to estimate 5-year and 8-year OS probabilities in malignant renal tumors (**A**). Calibration curves of the nomogram for 8-year OS (**B**) and 5-year OS (**C**) in the internal validation cohort. The gray diagonal line indicates perfect agreement between predicted and observed survival.

A nomogram was constructed based on the AI-aggressiveness score, TNM stage, ISUP grade, necrosis, and sarcomatoid differentiation to predict postoperative overall survival for malignant renal tumors (Figure 4A). This nomogram can be utilized to provide personalized estimates of a patient’s 5-year and 8-year OS probabilities. Calibration curves derived from the internal validation cohort demonstrated that the model possessed good fitness for both 5-year and 8-year OS predictions, with estimated probabilities generally aligning with the actual observations, indicating robust calibration performance (Figure 4B,C).

**Trends in small renal mass and solid tumor subgroups**

In the small renal mass subgroup (tumor size ≤ 4 cm), radiomics-predicted aggressiveness showed endpoint-dependent prognostic performance (Figure S2). In the internal validation cohort, significant survival separation was observed for DSS (*p* = 0.019, HR = 8.80) and OS (*p* = 0.007, HR = 4.06), whereas the difference in RFS was not statistically significant (*p* = 0.371, HR = 1.81). In the external test cohort, OS remained significantly different between the two radiomics-predicted groups (*p* = 0.027, HR = 3.96), whereas DSS (*p* = 0.061, HR = 4.17) and RFS (*p* = 0.330, HR = 2.09) did not reach statistical significance. In contrast, among the solid renal tumors, radiomics-predicted aggressiveness consistently stratified DSS, RFS, and OS in both the internal validation and external test cohorts (all *p* < 0.001; Figure S3). These findings suggest that the prognostic performance of the radiomics-predicted phenotype is particularly consistent in solid tumors, whereas its prognostic value in small renal masses varies according to the survival endpoint.

## 4. Discussion

This study developed and externally validated an SVM radiomics model based on multicenter preoperative multiphase CT data to differentiate indolent from aggressive renal tumors, and further demonstrated that model-predicted aggressiveness was closely associated with postoperative survival outcomes. Our principal findings indicate that the model maintained stable discriminative performance across both the validation and independent external test cohorts; radiomics-predicted aggressiveness was not only significantly associated with poorer RFS, DSS, and OS endpoints, but also retained independent prognostic value after multivariate adjustment. Furthermore, a nomogram integrating the AI-aggressiveness score, TNM stage, ISUP grade, necrosis, and sarcomatoid differentiation was developed to individualize 5-year and 8-year OS probability estimates. Collectively, these results support the premise that “radiographic aggressiveness” is not merely a preoperative classification label but also a risk-stratification phenotype with potential clinical interpretability.

Compared to previous studies that predominantly focused on identifying the ccRCC subtype, predicting nuclear grade, or modeling a single outcome, this research places greater emphasis on the comprehensive “indolent/aggressive” endpoint, which aligns more closely with real-world clinical decision-making scenarios. In recent years, reviews concerning radiomics and AI in RCC have generally concluded that imaging features can provide intratumoral heterogeneity information beyond visual assessment, demonstrating growing value in subtyping, grading, prognosis, and explaining underlying biological mechanisms [15,16,17]. Building upon this, multiple recent multicenter studies have extended radiomics or deep learning approaches to outcome prediction and survival stratification. For instance, a deep learning radiomics nomogram based on CT was applied for postoperative outcome prediction in ccRCC, outperforming traditional clinical models using multicenter data [18]; other studies constructed CT radiomics models specifically for postoperative survival prediction [19,20]. Therefore, the incremental value of our study lies in the fact that building upon the established indolent/aggressive pathological framework, we did not merely conclude that “imaging can identify high-risk lesions” but further bridged this phenotype with postoperative recurrence and overall survival, enhancing its clinical applicability through C-index evaluations and nomogram formulation.

The clinical significance of our findings warrants particular emphasis within the context of small renal masses and risk-adapted treatment strategies. Currently, the management of SRMs has transitioned from a “detect and resect” paradigm to an individualized pathway that emphasizes balancing tumor biology, renal function preservation, and the patient’s overall condition [21,22]. Recent literature on SRM management indicates that active surveillance is not inferior to immediate intervention for specific cT1a lesions, and differences in overall survival often reflect patients’ competing mortality risks rather than tumor progression itself [22]. Concurrently, evidence supporting renal mass biopsy continues to accumulate: large-sample registry studies, systematic reviews, and real-world cohorts all suggest that RMB possesses high diagnostic efficacy, can reduce unnecessary surgeries for benign or indolent lesions, and substantially impacts treatment pathway selection [23,24,25]. In this context, the preoperative non-invasive radiomics model developed in this study is best conceptualized as a tool complementary to, rather than a replacement for, biopsy. By providing probability estimates of aggressiveness at the initial imaging phase, it can help filter patients more suitable for active surveillance, nephron-sparing surgery, thermal ablation, or further pathological sampling, shifting clinical decision-making closer to a “risk-adapted” rather than a “morphology-driven” approach.

From a prognostic perspective, our results suggest that the radiomics aggressiveness score captures information that is not simply a surrogate for traditional variables such as TNM stage, WHO/ISUP grade, or necrosis, but may additionally reflect spatial tumor heterogeneity and deeper biological characteristics. Recently, numerous studies have explored this trajectory: some work has shown that radiomics models can effectively predict the WHO/ISUP grade of ccRCC [26], while others have linked Ki-67 expression status, prognostic risk, and imaging features, thereby enhancing the biological interpretability of the models [27]; other multicenter prognostic studies have revealed that interpretable machine learning radiomics models can identify the 5-year recurrence risk after surgery for non-metastatic ccRCC [28]. Furthermore, RCC imaging research is progressively shifting from “radiomics” to “radiogenomics”, emphasizing the associations between imaging traits, molecular pathways, the tumor microenvironment, and aggressive phenotypes [19]. Therefore, by utilizing the comprehensive “indolent/aggressive” phenotype as the target variable, our study is arguably more reflective of clinical scenarios than predicting nuclear grade alone, and possesses a greater capacity to encapsulate complex, heterogeneous biological information.

The nomogram presented in Figure 4 further bolsters the clinical translation potential of this study. In recent years, radiomics research in the RCC domain has been transitioning from merely reporting AUC values to addressing whether these models can be translated into interpretable tools for individualized decision-making [15,18,20]. In the present analysis, we integrated the AI-aggressiveness score with TNM stage, ISUP grade, necrosis, and sarcomatoid differentiation to construct an OS prediction nomogram, which exhibited favorable calibration performance in the internal validation cohort. This outcome is consistent with the recent trend of prognosis-targeted radiomics nomogram research, which aims to improve model readability and clinical utility by integrating imaging features with clinicopathological variables [18]. Importantly, sarcomatoid differentiation retained independent prognostic significance in the updated multivariate Cox models for RFS, OS, and DSS, further supporting its inclusion in the integrated prognostic model together with TNM stage, WHO/ISUP grade, and necrosis. Future integration with external calibration and decision curve analysis would make the clinical application of this nomogram even more compelling.

This study also has several notable strengths. First, the models were developed using a multicenter real-world database and evaluated with an independent external test cohort, thereby enhancing generalizability. Second, we systematically evaluated the prognostic value of the radiomics aggressive phenotype using Kaplan–Meier curves, Cox regression, C-index comparisons, and nomogram construction, extending beyond a mere assessment of classification performance. Third, the inclusion of clinically critical subgroups, such as small renal masses and solid tumors, facilitated a preliminary observation of the model’s applicability in more specific contexts. Compared to many single-center studies or those limited to a single pathological metric, this integrated “classification + prognosis + individual prediction” analytical approach aligns better with the current trajectory of AI in RCC imaging transitioning from technical validation to clinical translation.

Naturally, this study carries certain limitations. First, given the retrospective multicenter design, variations in scanning parameters, imaging protocols, and patient selection across centers may still influence feature stability and model transferability despite external testing, a common challenge in current RCC radiomics research [29]. Second, Figure 4 currently only illustrates calibration performance within the internal validation cohort, lacking external calibration and clinical net benefit analyses; thus, the generalizability of the nomogram requires further validation. Third, although this study sought to associate radiographic aggressiveness with survival, it did not incorporate molecular subtyping, immune microenvironment profiling, or more direct radiogenomic evidence. Incorporating digital pathology images, genomic, or transcriptomic data in future studies could further enhance the model’s interpretability and robustness [29].

## 5. Conclusions

In summary, this study developed and externally validated an SVM-based CT radiomics model to differentiate indolent from aggressive renal tumors and stratify postoperative prognosis. The model-predicted aggressiveness retained independent prognostic significance after multivariate adjustment, suggesting that it could serve as a valuable adjunct to TNM staging, ISUP grading, and traditional adverse pathological features. Considering that the contemporary management of localized renal tumors increasingly emphasizes individualization, renal preservation, and the avoidance of overtreatment, such non-invasive, quantifiable imaging tools applicable for preoperative risk assessment hold clear translational potential. Future prospective, multicenter studies with standardized protocols are warranted to further validate the robustness and clinical implementability of this approach.

## Figures and Tables

**Table 1:** Baseline characteristics of the study cohorts.

Feature	Training Cohort N = 1840	Internal Validation Cohort N = 789	External Test Cohort N = 778	Overall N = 3407
**Age, years, median (IQR)**	58.0 (50.0–66.0)	57.0 (50.0–65.0)	56.0 (47.0–64.0)	58.0 (50.0–65.0)
**Male sex, n (%)**	1229 (66.8%)	520 (65.9%)	534 (68.6%)	2283 (67.0%)
**Surgical procedure**				
Partial nephrectomy	788 (42.8%)	354 (44.9%)	538 (69.2%)	1680 (49.3%)
Radical nephrectomy	1052 (57.2%)	435 (55.1%)	240 (30.8%)	1727 (50.7%)
**Histologic aggressiveness**				
Indolent	1391 (75.6%)	597 (75.7%)	598 (76.9%)	2586 (75.9%)
Aggressive	449 (24.4%)	192 (24.3%)	180 (23.1%)	821 (24.1%)
**Detailed histologic subtype, n (%)**				
Clear cell RCC	1485 (80.7%)	629 (79.7%)	645 (82.9%)	2759 (81.0%)
Chromophobe RCC	108 (5.9%)	53 (6.7%)	43 (5.5%)	204 (6.0%)
Papillary RCC	118 (6.4%)	53 (6.7%)	32 (4.1%)	203 (6.0%)
Multilocular cystic renal neoplasm of low malignant potential	22 (1.2%)	8 (1.0%)	8 (1.0%)	38 (1.1%)
Epithelioid angiomyolipoma	22 (1.2%)	10 (1.3%)	3 (0.4%)	35 (1.0%)
Clear cell papillary renal cell tumor	18 (1.0%)	5 (0.6%)	6 (0.8%)	29 (0.9%)
TFE3-rearranged RCC	16 (0.9%)	7 (0.9%)	4 (0.5%)	27 (0.8%)
RCC, NOS	12 (0.7%)	1 (0.1%)	10 (1.3%)	23 (0.7%)
Low-grade malignant renal neoplasm	7 (0.4%)	4 (0.5%)	11 (1.4%)	22 (0.6%)
Renal sarcoma	10 (0.5%)	6 (0.8%)	2 (0.3%)	18 (0.5%)
Mucinous tubular and spindle cell carcinoma	6 (0.3%)	5 (0.6%)	5 (0.6%)	16 (0.5%)
High-grade malignant renal neoplasm	0 (0.0%)	2 (0.3%)	5 (0.6%)	7 (0.2%)
Eosinophilic solid and cystic RCC	3 (0.2%)	0 (0.0%)	4 (0.5%)	7 (0.2%)
Other oncocytic renal neoplasm	3 (0.2%)	3 (0.4%)	0 (0.0%)	6 (0.2%)
TFEB-altered RCC	4 (0.2%)	0 (0.0%)	0 (0.0%)	4 (0.1%)
Solitary fibrous tumor	2 (0.1%)	1 (0.1%)	0 (0.0%)	3 (0.1%)
Nephroblastoma	1 (0.1%)	0 (0.0%)	0 (0.0%)	1 (0.0%)
Tubulocystic RCC	0 (0.0%)	1 (0.1%)	0 (0.0%)	1 (0.0%)
Well-differentiated neuroendocrine tumor	1 (0.1%)	0 (0.0%)	0 (0.0%)	1 (0.0%)
Succinate dehydrogenase-deficient RCC	0 (0.0%)	1 (0.1%)	0 (0.0%)	1 (0.0%)
Inflammatory myofibroblastic tumor	1 (0.1%)	0 (0.0%)	0 (0.0%)	1 (0.0%)
Collecting duct carcinoma	1 (0.1%)	0 (0.0%)	0 (0.0%)	1 (0.0%)
**Tumor size, cm, median (IQR)**	3.5 (2.5–5.0)	3.5 (2.5–5.0)	3.2 (2.4–4.5)	3.5 (2.5–5.0)
**Small renal mass (≤4 cm), n (%)**	1167 (63.4%)	510 (64.6%)	551 (70.8%)	2228 (65.4%)
**Imaging appearance**				
Solid	1539 (83.6%)	666 (84.4%)	682 (87.7%)	2887 (84.7%)
Cystic	301 (16.4%)	123 (15.6%)	96 (12.3%)	520 (15.3%)
**T stage, n (%)**				
T1	1528 (83.0%)	652 (82.6%)	691 (88.8%)	2871 (84.3%)
T2	152 (8.3%)	58 (7.4%)	52 (6.7%)	262 (7.7%)
T3	160 (8.7%)	79 (10.0%)	35 (4.5%)	274 (8.0%)
**AJCC stage, n (%)**				
Stage I	1532 (83.3%)	655 (83.0%)	691 (88.8%)	2878 (84.5%)
Stage II	149 (8.1%)	57 (7.2%)	51 (6.6%)	257 (7.5%)
Stage III	159 (8.6%)	77 (9.8%)	36 (4.6%)	272 (8.0%)
**WHO/ISUP grade, n (%)**				
Low (1 & 2)	1334 (72.5%)	579 (73.4%)	533 (68.5%)	2446 (71.8%)
High (3 & 4)	265 (14.4%)	100 (12.7%)	136 (17.5%)	501 (14.7%)
Unknown/Not applicable	241 (13.1%)	110 (13.9%)	109 (14.0%)	460 (13.5%)

**Note:** Data are presented as median (interquartile range, IQR) or number (percentage), as appropriate. Small renal mass was defined as tumor size ≤ 4 cm. WHO/ISUP grade was categorized as low (grades 1–2), high (grades 3–4), or unknown/not applicable when unavailable or not applicable.

**Table 2:** Classification performance of the radiomics model for discriminating indolent and aggressive renal tumors across different cohorts.

Metrics	Training Cohort	Internal Validation Cohort	External Test Cohort
AUC (95% CI)	0.723 (0.699–0.752)	0.740 (0.697–0.776)	0.763 (0.730–0.800)
Accuracy (95% CI)	0.596 (0.578–0.618)	0.624 (0.589–0.660)	0.746 (0.722–0.778)
Sensitivity (95% CI)	0.780 (0.746–0.815)	0.812 (0.750–0.869)	0.617 (0.559–0.685)
Specificity (95% CI)	0.537 (0.512–0.562)	0.563 (0.527–0.605)	0.784 (0.752–0.820)
F1 score (95% CI)	0.485 (0.451–0.510)	0.512 (0.447–0.559)	0.529 (0.477–0.593)

**Abbreviations:** AUC, area under the receiver operating characteristic curve; CI, confidence interval; F1 score, harmonic mean of precision and recall.

**Table 3:** Univariate and multivariate Cox regression analyses for recurrence-free survival in malignant renal tumors.

Variables	Univariate Analysis			Multivariate Analysis		
	Hazard Ratio	95% CI	*p*-Value	Hazard Ratio	95% CI	*p*-Value
**Radiomics-predicted aggressiveness**			<0.001			<0.001
Aggressive vs. indolent	6.565	4.353–9.902	<0.001	3.656	2.362–5.659	<0.001
**TNM stage**			<0.001			<0.001
II vs. I	4.397	2.891–6.687	<0.001	1.784	1.138–2.797	0.012
III vs. I	8.546	6.089–11.994	<0.001	3.885	2.702–5.587	<0.001
**WHO/ISUP grade**			<0.001			<0.001
II vs. I	1.977	0.721–5.423	0.185	1.750	0.638–4.803	0.277
III vs. I	10.151	3.696–27.881	<0.001	4.771	1.712–13.295	0.003
IV vs. I	29.677	9.289–94.818	<0.001	5.225	1.458–18.732	0.011
Unknown/Not applicable vs. I	3.893	1.362–11.126	0.011	1.930	0.654–5.699	0.234
**Necrosis**			<0.001			0.031
Present vs. absent	4.741	3.456–6.506	<0.001	1.497	1.037–2.161	0.031
**Sarcomatoid differentiation**			<0.001			<0.001
Present vs. absent	13.234	7.995–21.906	<0.001	4.414	2.132–9.136	<0.001

**Abbreviations:** RFS, recurrence-free survival; CI, confidence interval; WHO/ISUP, World Health Organization/International Society of Urological Pathology. **Note:** Only renal tumors with available follow-up were included in the survival analyses. Cox proportional hazards models used Breslow handling of tied event times. WHO/ISUP grade I was the reference category, and Unknown/Not applicable was retained as a separate category. Any non-zero recorded code for necrosis or sarcomatoid differentiation was treated as present.

## Data Availability

The original clinical and imaging datasets contain sensitive patient information and are governed by the data management regulations of participating hospitals. Therefore, the data cannot be publicly released. De-identified research data can be provided to qualified researchers upon a formal request to the corresponding author.

## Supplementary Materials

The following supporting information can be downloaded at: https://www.mdpi.com/article/10.3390/bioengineering13091084/s1; Figure S1: Flowchart of patient enrollment and cohort allocation; Figure S2: Kaplan–Meier survival analyses in small renal masses; Figure S3: Kaplan–Meier survival analyses in solid renal tumors; Table S1: Definitions of the extracted radiomic features ranked in descending order by their LASSO coefficients; Table S2: Univariate and multivariate Cox regression analyses for overall survival in malignant renal tumors; Table S3: Univariate and multivariate Cox regression analyses for disease-specific survival in malignant renal tumors.

## Abbreviations

The following abbreviations are used in this manuscript:

AUC	Area under the receiver operating characteristic curve
ccRCC	Clear cell renal cell carcinoma
DSC	Dice similarity coefficient
DSS	Disease-specific survival
LASSO	Least absolute shrinkage and selection operator
OS	Overall survival
pRCC	Papillary renal cell carcinoma
RCC	Renal cell carcinoma
RFS	Recurrence-free survival
RMB	Renal mass biopsy
ROC	Receiver operating characteristic
ROI	Region of interest
SRMs	Small renal masses
SVM	Support vector machine
