# Supplementary material for: Noninvasive Preoperative Stratification of Renal Tumors for Aggressiveness and Prognosis
Source: Bioengineering (Basel). 2026 Sep 19;13(9):1084. doi: 10.3390/bioengineering13091084 (PMC13603619; doi:10.3390/bioengineering13091084)
Supplement: Supplementary file 1 [file bioengineering-13-01084-s001.zip › Bioengineering_supplementary_tables_final_recalculated.pdf]

Supplementary Table S1. Definitions of the extracted radiomic features ranked in descending order by their LASSO coefficients.

No	The Name of RF	The defination of RF
1	N_original_shape2D_MajorAxisLength	Major Axis Length of the tumor in N phase
2	V_original_firstorder_RootMeanSquared	Root Mean Squared Intensity in V phase
3	V_original_firstorder_Energy	Energy of Intensity values in V phase
4	V_original_glcm_DifferenceEntropy	Difference Entropy of GLCM in V phase
5	N_original_firstorder_90Percentile	90th percentile of Intensity in N phase
6	A_original_firstorder_Kurtosis	Kurtosis of Intensity in A phase
7	V_original_glszm_ZoneVariance	Zone Variance of GLSZM in V phase
8	A_original_glcm_Idn	Inverse Difference Normalized of GLCM in A phase
9	A_original_firstorder_90Percentile	90th percentile of Intensity in A phase
10	N_original_shape2D_Elongation	Elongation of the tumor in N phase

Supplementary Table S2. Univariate and multivariate Cox regression analyses for overall survival in malignant renal tumors

**Variables**	**Univariate analysis**			**Multivariate analysis**		
	**Hazard ratio**	**95% CI**	**p-value**	**Hazard ratio**	**95% CI**	**p-value**
**Radiomics-predicted aggressiveness**			<0.001			<0.001
Aggressive vs indolent	3.419	2.428-4.816	<0.001	1.981	1.361-2.882	<0.001
**TNM stage**			<0.001			<0.001
II vs I	3.531	2.322-5.368	<0.001	1.790	1.136-2.822	0.012
III vs I	5.770	4.048-8.224	<0.001	3.130	2.133-4.594	<0.001
**WHO/ISUP grade**			<0.001			<0.001
II vs I	1.749	0.707-4.326	0.226	1.596	0.645-3.950	0.312
III vs I	6.906	2.764-17.255	<0.001	3.777	1.486-9.605	0.005
IV vs I	30.250	10.482-87.297	<0.001	8.843	2.743-28.503	<0.001
Unknown/Not applicable vs I	3.148	1.212-8.175	0.019	1.955	0.737-5.191	0.178

Variables	Univariate analysis			Multivariate analysis		
	Hazard ratio	95% CI	p-value	Hazard ratio	95% CI	p-value
**Necrosis**			<0.001			0.015
Present vs absent	4.018	2.908-5.552	<0.001	1.598	1.097-2.327	0.015
**Sarcomatoid differentiation**			<0.001			0.017
Present vs absent	9.527	5.508-16.479	<0.001	2.458	1.171-5.158	0.017

Abbreviations: OS, overall survival; CI, confidence interval; WHO/ISUP, World Health Organization/International Society of Urological Pathology.

Note: Only renal tumors with available follow-up were included in the survival analyses. Cox proportional hazards models used Breslow handling of tied event times. WHO/ISUP grade I was the reference category, and Unknown/Not applicable was retained as a separate category. Any non-zero recorded code for necrosis or sarcomatoid differentiation was treated as present.

Supplementary Table S3. Univariate and multivariate Cox regression analyses for disease-specific survival in malignant renal tumors

Variables	Univariate analysis			Multivariate analysis		
	Hazard ratio	95% CI	p-value	Hazard ratio	95% CI	p-value
**Radiomics-predicted aggressiveness**			<0.001			<0.001
Aggressive vs indolent	7.444	4.392-12.616	<0.001	3.673	2.095-6.439	<0.001
**TNM stage**			<0.001			<0.001
II vs I	5.579	3.419-9.102	<0.001	2.113	1.251-3.569	0.005
III vs I	10.245	6.811-15.410	<0.001	4.449	2.877-6.878	<0.001
**WHO/ISUP grade**			<0.001			<0.001
II vs I	1.418	0.437-4.606	0.561	1.241	0.382-4.035	0.720
III vs I	9.727	3.027-31.257	<0.001	4.186	1.278-13.717	0.018

Variables	Univariate analysis			Multivariate analysis		
	Hazard ratio	95% CI	p-value	Hazard ratio	95% CI	p-value
IV vs I	47.473	13.207-170.642	<0.001	10.933	2.751-43.457	<0.001
Unknown/Not applicable vs I	3.843	1.142-12.934	0.030	1.892	0.543-6.590	0.317
**Necrosis**			<0.001			0.024
Present vs absent	5.865	4.047-8.500	<0.001	1.638	1.066-2.517	0.024
**Sarcomatoid differentiation**			<0.001			0.007
Present vs absent	14.162	8.091-24.790	<0.001	2.884	1.333-6.241	0.007

Abbreviations: DSS, disease-specific survival; CI, confidence interval; WHO/ISUP, World Health Organization/International Society of Urological Pathology.

Note: Only renal tumors with available follow-up were included in the survival analyses. Cox proportional hazards models used Breslow handling of tied event times. WHO/ISUP grade I was the reference category, and Unknown/Not applicable was retained as a separate category. Any non-zero recorded code for necrosis or sarcomatoid differentiation was treated as present.
